# Recurrent Pericarditis in a Patient With Ankylosing Spondylitis: A Report of a Rare Case

**DOI:** 10.7759/cureus.96181

**Published:** 2025-11-05

**Authors:** Constantin Sitari, Nicolae Sitari, Isabel Rodrigues, Rosario Blanco Sáez, Alexandra Martins

**Affiliations:** 1 Department of Internal Medicine, Unidade Local de Saúde do Algarve - Hospital de Faro, Faro, PRT; 2 Department of Cardiology, Institutul de Boli Cardiovasculare "Prof. Dr. George I. M. Georgescu”, Iași, ROU

**Keywords:** ankylosing spondylitis, anti-tnf, echocardiography, electrocardiography, pericardial effusion, pericarditis, recurrent pericarditis

## Abstract

Only a small subset of patients with ankylosing spondylitis (AS) develop cardiac complications, and pericardial inflammation is particularly uncommon. We report a 31-year-old human leukocyte antigen B27 (HLA-B27)-positive man with AS diagnosed in 2018 who, after discontinuing golimumab in 2021, experienced two episodes of acute pericarditis in 2025. In March, he presented with fever and chest pain. The electrocardiogram (ECG) showed diffuse, mild concave ST-segment elevation; chest computed tomography (CT) demonstrated a circumferential pericardial effusion measuring 24 mm with bilateral pleural effusions, consistent with polyserositis, and transthoracic echocardiography (TTE) confirmed a 15-17 mm fibrinous effusion without tamponade. He improved with colchicine and a corticosteroid taper. In August, he returned with pleuritic chest pain; the ECG revealed sinus tachycardia with diffuse ST elevation and PR-segment depression, and echocardiography showed a thin residual effusion. Bacterial and viral infections (including multiplex testing for respiratory and enteroviral pathogens and serology for cytomegalovirus, Epstein-Barr virus, and parvovirus B19), HIV, hepatitis B and C, and autoimmune causes were systematically excluded, and there were no clinical or imaging features to suggest malignancy. Taken together, the relapse and overall context favored attribution to AS-related inflammation. This case underscores the need to keep pericarditis in mind when patients with AS present with chest pain and to align pericarditis therapy with control of the underlying disease.

## Introduction

Ankylosing spondylitis (AS) is a chronic axial spondyloarthritis frequently associated with human leukocyte antigen B27 (HLA-B27) that, beyond axial disease, can involve extra-articular organs, including the heart; cardiac involvement most often affects the aortic root or valves and the conduction system, whereas pericardial disease is uncommon [1]. Prevalence depends on ascertainment; in a Moroccan cross-sectional cohort with systematic electrocardiogram and transthoracic echocardiography screening, 13.3% of spondyloarthritis patients had cardiac involvement, and approximately 3.3% had pericarditis [2]. Reported clinical presentations of AS-related pericardial disease range from small effusions to life-threatening tamponade [3-5]. Polyserositis with concomitant pleural and pericardial effusions has also been described and usually prompts broad etiologic exclusion before attribution to AS [4,6]. Inflammation-driven pathology in AS, implicating the aortic root, valvular structures, and the conduction system, provides a plausible substrate for occasional pericardial involvement as part of the same inflammatory milieu [1]. Management of axial spondyloarthritis follows the ASAS-EULAR 2022 update, which recommends nonsteroidal anti-inflammatory drugs (NSAIDs) as first-line therapy and escalation to biologic or targeted synthetic agents, such as tumor necrosis factor (TNF) or interleukin-17 (IL-17) inhibitors and Janus kinase inhibitors, based on disease activity and phenotype [7]. Case-based experience also suggests that controlling the underlying axial disease with TNF inhibitors can coincide with improvement in severe pericardial involvement, although evidence remains limited [3]. Pharmacovigilance analyses have identified a disproportionate reporting signal of pericarditis with several biologic disease-modifying antirheumatic drugs used in AS; while this does not establish causality, it supports individualized risk-benefit assessment and careful appraisal of temporality when symptoms arise [8]. Broader challenges include under-recognition and heterogeneity of cardiovascular comorbidity in axial spondyloarthritis and potential sex-related differences in risk, emphasizing coordinated rheumatology-cardiology follow-up and durable inflammatory control [9,10]. Against this background, we report recurrent acute pericarditis with concomitant pleural effusions in a young HLA-B27-positive man with AS, an uncommon but clinically consequential presentation that underscores systematic etiologic exclusion and alignment of pericarditis therapy with optimization of axial disease control [4,6].

## Case presentation

A 31-year-old HLA-B27-positive man with AS diagnosed in 2018 had been treated with golimumab until 2021, when routine follow-up was discontinued.

**Figure 1:**
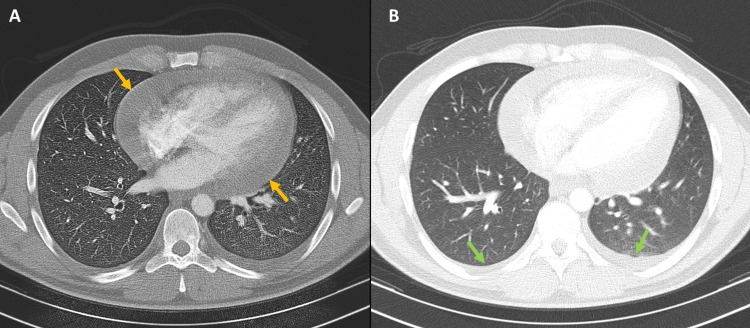
Chest computed tomography (March 9, 2025), axial images. (A) Mediastinal window showing a circumferential pericardial effusion (yellow arrows). (B) Lung window demonstrating bilateral pleural effusions (green arrows).

**Figure 2:**
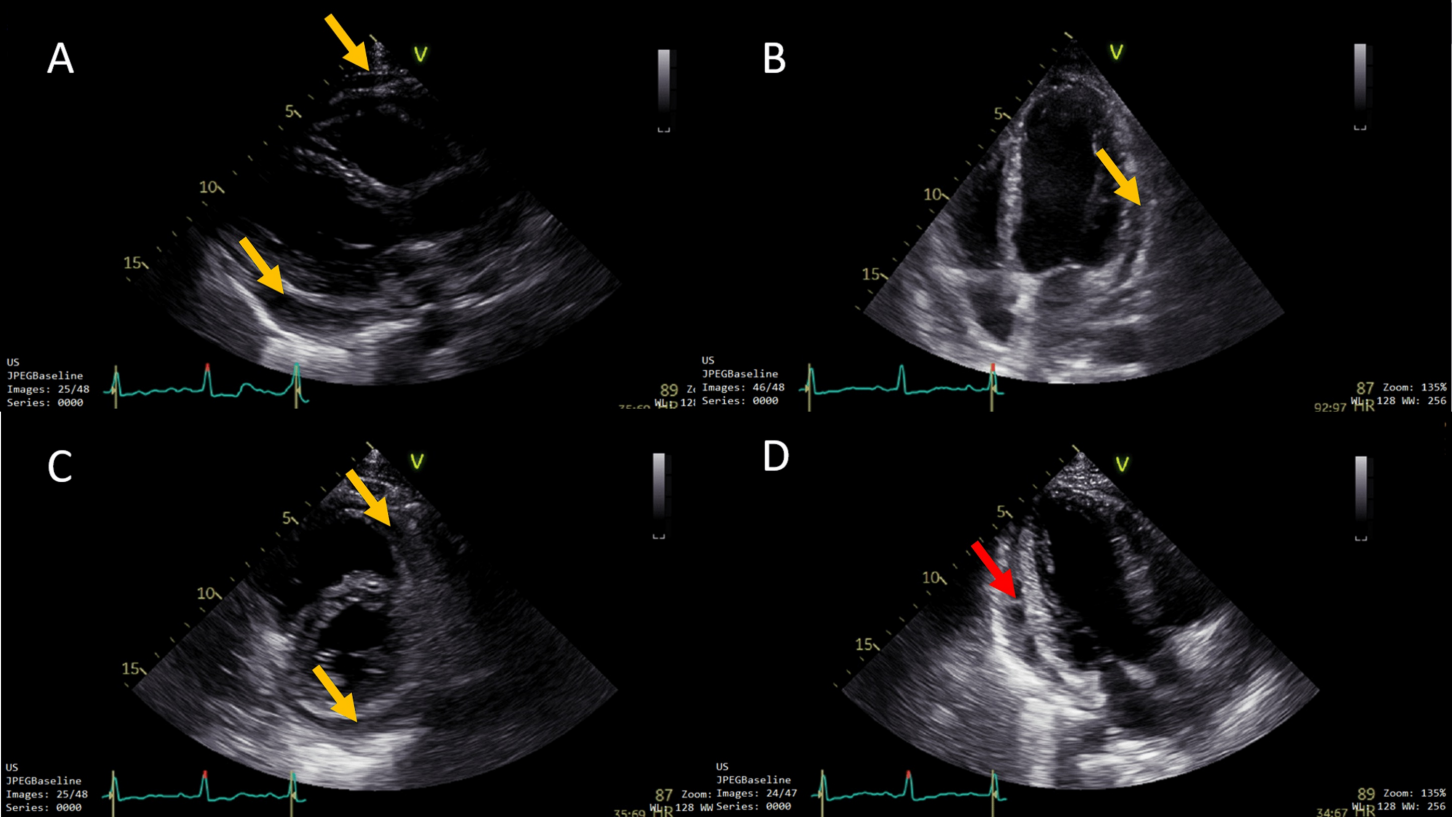
Transthoracic echocardiography (March 12, 2025). Images show evidence of pericardial effusion (yellow arrows) in the parasternal long-axis view (A), apical four-chamber view (B), parasternal short-axis view (C), and fibrin strands in the apical three-chamber view (D) (red arrow).

**Figure 3:**
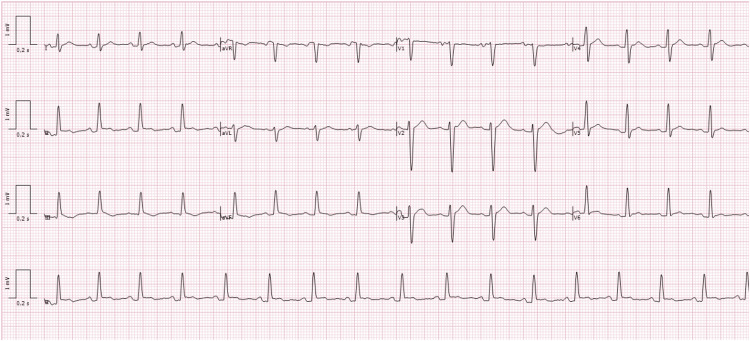
Twelve-lead electrocardiogram (March 9, 2025). The ECG shows a regular sinus rhythm at a ventricular rate of 98 beats per minute with discrete ST-segment elevation and PR-segment depression in leads I, II, and V2-V6, consistent with stage 1 (acute phase) of pericarditis evolution.

On March 9, 2025, he arrived with a 39°C fever, oppressive chest pain, and dyspnea. He also reported inflammatory low back pain, with physical examination showing right-sided sacroiliac tenderness and a positive Patrick (FABER) test, suggestive of active axial disease. Vitals and laboratory data are provided in Table 1, notably leukocytes 13.5 × 10^9^/L, C-reactive protein (CRP) 130 mg/L, and high-sensitivity troponin (hs-troponin) 4.7 ng/L. Blood and urine cultures were negative. Nasopharyngeal multiplex testing was negative for enteroviruses/Coxsackievirus, adenovirus, influenza, and severe acute respiratory syndrome coronavirus 2 (SARS‑CoV‑2). Serology indicated prior immunity to cytomegalovirus (IgG positive, IgM negative) and Epstein-Barr virus (IgG positive, IgM negative), was negative for parvovirus B19, and was negative for HIV, hepatitis B virus (HBV), and hepatitis C virus (HCV). Chest CT showed a circumferential pericardial effusion measuring 24 mm with bilateral pleural effusions (Figure 1), and a transthoracic echocardiogram (TTE) confirmed a 15-17 mm fibrinous circumferential effusion without hemodynamic compromise (Figure 2). The ECG (March 9, 2025) demonstrated a regular sinus rhythm at 98 bpm with discrete ST-segment elevation and PR-segment depression in leads I, II, and V2-V6 (Figure 3). He was hospitalized and treated with colchicine 0.5 mg twice daily and prednisone 40 mg/day with a taper (40 mg for one week, 20 mg for one week, 10 mg for one week, then maintained at 5 mg daily until the autoimmune clinic follow-up, as previously scheduled); empiric antibiotics were discontinued after negative cultures. He improved and was discharged on day 4. At cardiology follow-up on May 5, 2025, laboratory tests demonstrated normalization of inflammatory markers (Table 1) and recovery of hematologic and biochemical parameters, confirming clinical remission before the second episode.

**Table 1:** Vitals and routine laboratory results across episodes. All measurements are reported with their respective units; reference ranges reflect institutional laboratory intervals. SpO_2_: peripheral oxygen saturation; CRP: C-reactive protein; hs (troponin): high-sensitivity; ESR: erythrocyte sedimentation rate; ALT: alanine aminotransferase; AST: aspartate aminotransferase; INR: international normalized ratio; LDL: low-density lipoprotein; HDL: high-density lipoprotein; NT-proBNP: N-terminal pro-B-type natriuretic peptide ^*^ two distinct observations within the same encounter; ^†^ test obtained on March 11, 2025, during the same admission as episode 1.

Parameter	Episode 1 (March 9, 2025)	Cardiology follow-up (May 5, 2025)	Episode 2 (August 3, 2025)	Autoimmunity follow-up (August 28, 2025)	Reference range	Units
Temperature (tympanic)	37.0	-	37.4	-	36.0-37.5	°C
Heart rate	113	-	114/73*	-	60-100	bpm
Blood pressure	144/90	-	123/65	-	90-140/60-90	mmHg
SpO_2_ (room air)	100	-	98	-	≥95	%
White blood cells	13.5	9.7	12.5	9.7	4.0-10.0	×10^9^/L
Neutrophils (absolute)	10.2	5.9	9.4	5.0	2.0-7.0	×10^9^/L
Hemoglobin	132	144	124	143	130-170	g/L
Platelets	323	219	254	206	150-400	×10^9^/L
CRP	130	8	208	-	<5	mg/L
ESR	85^†^	-	-	12	<20	mm/h
Troponin-T (hs)	4.7	10.1	6.3	-	≤14	ng/L
NT-proBNP	56	-	-	-	<450	pg/mL
Glucose	121	85	112	86	<110 (fasting)	mg/dL
Sodium	138	139	137	140	136-144	mmol/L
Potassium	5.1	4.5	4.3	4.7	3.3-5.1	mmol/L
Chloride	101	101	97	101	101-111	mmol/L
Creatinine	0.9	0.9	0.9	-	0.7-1.3	mg/dL
Blood urea nitrogen	16	15	12	11	8.9-20.6	mg/dL
ALT	34	21	-	25	<55	U/L
AST	34	22	-	-	5-34	U/L
Total bilirubin	0.50	0.30	-	-	0.20-1.20	mg/dL
INR	1.2	0.9	1.1	-	-	-
LDL-cholesterol	-	-	-	138	<100	mg/dL
HDL-cholesterol	-	-	-	40	>40	mg/dL
Triglycerides	-	-	-	175	<150	mg/dL
Total cholesterol	-	-	-	196	<200	mg/dL

**Figure 4:**
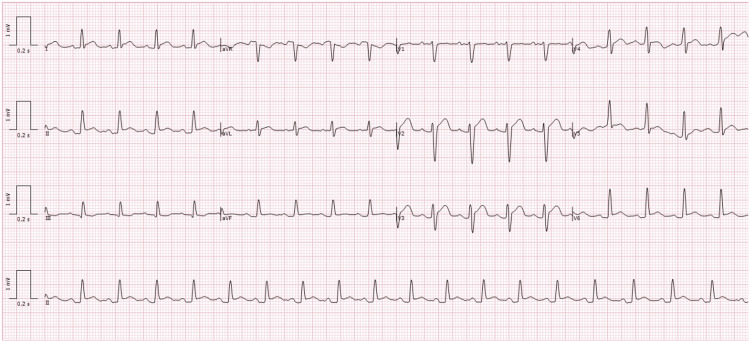
Twelve-lead electrocardiogram (August 3, 2025). The ECG shows sinus tachycardia at 114 beats per minute with widespread concave ST-segment elevation and PR-segment depression in leads I, II, III, aVL, aVF, and V2-V6, ST-segment depression and PR-segment elevation in aVR, consistent with stage 1 (acute phase), showing a more extensive inflammatory involvement than during the first episode.

On August 3, 2025, he re-presented with pleuritic chest pain and low-grade fever (maximum 37.5°C), while still on prednisone 5 mg daily from the taper regimen initiated after the first episode. Vitals and laboratories are again summarized in Table 1, with leukocytes 12.5 × 10^9^/L, CRP 208 mg/L, and troponin 6.3 ng/L. ECG revealed sinus tachycardia at 114 bpm with widespread concave ST-segment elevation and PR segment depression in leads I, II, III, aVL, aVF, and V2-V6, with reciprocal ST-segment depression and PR segment elevation in aVR (Figure 4). TTE showed only a thin residual effusion without hemodynamic impact. He received aspirin 1 g intravenously followed by a six-week oral taper and colchicine 0.5 mg twice daily for three months, with a favorable course.

At the autoimmunity clinic follow-up, with infectious and autoimmune causes excluded and no clinical or imaging suggestion of malignancy, restarting biologic therapy (golimumab) was planned; prednisone 5 mg/day was maintained.

## Discussion

Our patient’s large circumferential effusion at first presentation and subsequent recurrence fit the published spectrum of AS-related pericardial disease, which spans from small effusions to frank tamponade [3-5]. Coexisting pleural effusions, that is, polyserositis, are documented in AS and typically respond to anti-inflammatory therapy once infection, malignancy, and systemic autoimmune disease are excluded - an approach mirrored in our diagnostic work-up [4,6].

Although cardiac involvement in AS is often cited in single-digit percentages, real-world estimates vary with population and screening intensity; for example, a Moroccan cohort reported 13.3% overall cardiac involvement and approximately 3.3% pericarditis, supporting that clinically meaningful pericardial disease, while infrequent, does occur [2]. The patient’s age, HLA-B27 positivity, diffuse ST-segment elevation with PR depression, and imaging-confirmed effusion are concordant with patterns described in case reports of AS-related pericarditis [3-5].

Mechanistically, reviews emphasize inflammation-driven aortitis, valvular disease, and conduction disturbances in AS, with pericardial inflammation a rarer but biologically plausible extension of the same inflammatory milieu [1]. The presence of polyserositis in our case supports a systemic inflammatory driver after alternative etiologies were excluded, consistent with prior observations [4,6].

Cytokine-mediated inflammation involving IL-1/NLRP3, TNF-α, IL-6, and IL-17 pathways has been implicated in both spondyloarthritis and pericardial disease. IL-1β-driven inflammasome activation promotes vascular permeability and pericardial pain, whereas TNF-α and IL-6 sustain systemic inflammation, and IL-17 contributes to serosal fibrosis [11,12]. These overlapping cytokine networks may explain the occurrence of pericarditis as an extra-articular manifestation of AS and support the rationale for biologic anti-inflammatory therapy.

Initial management with high-dose aspirin or NSAIDs and colchicine, with a short corticosteroid taper in selected scenarios, resembles approaches seen in AS case literature for pericarditis control [3-5]. High-dose aspirin was selected as first-line anti-inflammatory therapy, consistent with the 2025 ESC guidelines for the management of myocarditis and pericarditis [13]. These guidelines recommend aspirin or an NSAID plus colchicine as first-line treatment for both acute and recurrent pericarditis (Class I, Level B), while corticosteroids are reserved for contraindications or treatment failure (Class III, Level C).

The temporal association between golimumab withdrawal and recurrence is compatible with an inflammatory rebound phenomenon. Studies in axial spondyloarthritis show that approximately 50% of patients relapse within six months of anti-TNF discontinuation and most within one year, reflecting restoration of TNF-α and downstream IL-23/IL-17 signaling once blockade is removed [14-17]. Recognition of this pattern informed our decision to restart biologic therapy. From the rheumatology perspective, re-initiating biologic therapy is consistent with the ASAS-EULAR 2022 escalation framework when disease activity persists or recurs [7]. Notably, case experience suggests that targeting the underlying axial disease with TNF inhibitors may coincide with improvement of severe pericardial involvement, which aligns with our patient’s favorable clinical course under anti-inflammatory therapy and planned biologic resumption [3]. Pericardiocentesis was not indicated, as the effusion remained hemodynamically stable without signs of tamponade and showed progressive resolution under anti-inflammatory therapy, consistent with ESC 2025 guidelines that support conservative management in stable cases without hemodynamic compromise [13].

Although our patient remains under early post-treatment observation, follow-up and recurrence prevention strategies for recurrent pericarditis in the context of systemic inflammatory diseases are well defined. The ESC 2025 guidelines and recent observational data recommend close monitoring for at least 12 months after remission, with clinical review at 1, 3, 6, and 12 months, serial CRP and ECG if chest pain recurs, and echocardiography at baseline and every 6-12 months or sooner if symptomatic [13]. To reduce recurrence risk, colchicine 0.5 mg twice daily for three to six months combined with gradual tapering of corticosteroids is the standard of care, while anti-TNF re-initiation is endorsed by ASAS-EULAR 2022 when systemic inflammation persists or recurs [7,12]. Future management should emphasize multidisciplinary cardiology-rheumatology coordination, sustained anti-inflammatory control, and individualized risk-benefit assessment of long-term biologic therapy.

Safety considerations include pharmacovigilance signals of disproportionate pericarditis reporting with multiple biologic disease-modifying antirheumatic drugs used in AS; while disproportionality does not establish causality, it supports individualized risk-benefit assessment and close monitoring of temporality if pericarditis emerges during therapy [8]. Beyond drug safety, axial spondyloarthritis carries broader cardiovascular risk and heterogeneity, potentially influenced by sex, supporting coordinated cardiology-rheumatology follow-up, risk-factor management, and sustained suppression of inflammatory activity [9,10].

## Conclusions

Clinicians should keep pericarditis on the diagnostic radar when patients with AS present with chest pain. Episodes may occasionally manifest as polyserositis with concurrent pericardial and pleural effusions. Before attributing the presentation to the underlying disease, infectious and autoimmune causes must be carefully ruled out through targeted testing and imaging.

Management should follow contemporary pericarditis care - NSAIDs or aspirin plus colchicine, with corticosteroids reserved for selected scenarios - and be aligned with sustained control of AS activity. In patients who have discontinued biologic therapy or exhibit recurrent episodes, reconsidering (re)introduction of a biologic agent is reasonable. Close follow-up is advisable to detect early signs of relapse and to titrate therapy accordingly.
